# Germline biomarkers predict toxicity to anti-PD1/PDL1 checkpoint therapy

**DOI:** 10.1136/jitc-2021-003625

**Published:** 2022-01-31

**Authors:** Joanne Weidhaas, Nicholas Marco, Aaron W Scheffler, Anusha Kalbasi, Kirk Wilenius, Emily Rietdorf, Jaya Gill, Mara Heilig, Caroline Desler, Robert K Chin, Tania Kaprealian, Susan McCloskey, Ann Raldow, Naga P Raja, Santosh Kesari, Jose Carrillo, Alexandra Drakaki, Mark Scholz, Donatello Telesca

**Affiliations:** 1 Department of Radiation Oncology, University of California Los Angeles, Los Angeles, California, USA; 2 Department of Biostatistics, UCLA, Los Angeles, California, USA; 3 Prostate Oncology Specialists, Marina Del Rey, California, USA; 4 Pacific Neuroscience Institute and Saint John’s Cancer Institute, Santa Monica, California, USA; 5 Appalachian Regional Healthcare, Hazard, Kentucky, USA; 6 Department of Urology, Medical Oncology, University of California Los Angeles, Los Angeles, California, USA

**Keywords:** autoimmunity, genetic markers

## Abstract

**Background:**

There is great interest in finding ways to identify patients who will develop toxicity to cancer therapies. This has become especially pressing in the era of immune therapy, where toxicity can be long-lasting and life-altering, and primarily comes in the form of immune-related adverse effects (irAEs). Treatment with the first drugs in this class, anti-programmed death 1 (anti-PD1)/programmed death-ligand 1 (PDL1) checkpoint therapies, results in grade 2 or higher irAEs in up to 25%–30% of patients, which occur most commonly within the first 6 months of treatment and can include arthralgias, rash, pruritus, pneumonitis, diarrhea and/or colitis, hepatitis, and endocrinopathies. We tested the hypothesis that germline microRNA pathway functional variants, known to predict altered systemic stress responses to cancer therapies, would predict irAEs in patients across cancer types.

**Methods:**

MicroRNA pathway variants were evaluated for an association with grade 2 or higher toxicity using four classifiers on 62 patients with melanoma, and then the panel’s performance was validated on 99 patients with other cancer types. Trained classifiers included classification trees, LASSO-regularized logistic regression, boosted trees, and random forests. Final performance measures were reported on the training set using leave-one-out cross validation and validated on held-out samples. The predicted probability of toxicity was evaluated for its association, if any, with response categories to anti-PD1/PDL1 therapy in the melanoma cohort.

**Results:**

A biomarker panel was identified that predicts toxicity with 80% accuracy (F1=0.76, area under the curve (AUC)=0.82) in the melanoma training cohort and 77.6% accuracy (F1=0.621, AUC=0.778) in the pan-cancer validation cohort. In the melanoma cohort, the predictive probability of toxicity was not associated with response categories to anti-PD1/PDL1 therapy (p=0.70). In the same cohort, the most significant biomarker of toxicity in *RAC1*, predicting a greater than ninefold increased risk of toxicity (p<0.001), was also not associated with response to anti-PD1/PDL1 therapy (p=0.151).

**Conclusions:**

A germline microRNA-based biomarker signature predicts grade 2 and higher irAEs to anti-PD1/PDL1 therapy, regardless of tumor type, in a pan-cancer manner. These findings represent an important step toward personalizing checkpoint therapy, the use of which is growing rapidly.

## Introduction

Checkpoint inhibitors are an exciting advance in the treatment of patients with cancer. Enthusiasm is justified, with response rates of over 20% in melanoma, non-small cell lung cancer (NSCLC), and genitourinary cancers (GU).^1^ However, a new form of toxicity resembling autoimmunity, where the immune system attacks host normal tissues, referred to as immune-related adverse events (irAEs), is a significant problem.^2 3^ irAEs from checkpoint therapy can affect the skin, liver, bowel, endocrine system, lung, heart, eyes, nerves, muscles, or the kidneys, and can be severe and even life-threatening. Grade 2 and higher irAEs, which require steroid treatment and a break from therapy, occur in 25%–30% of patients treated with single-agent checkpoint therapy and in up to 55% for combination immune therapies with increased severity.^2^ Currently there is no way to predict which patients will develop irAEs before starting treatment; thus, the strategy is to watch and wait after treatment initiation. As immune therapy is being broadly offered, even in the metastatic setting in tumor types where efficacy may not be fully determined,^1^ there is a growing need to identify patients at risk of such toxicities to allow a balanced discussion to guide treatment decisions.

While there are several tumor-based biomarkers predicting response to immune therapy, such as percent of tumor PDL1 expression^4^ and more recently tumor mutational burden and T cell-inflamed gene expression profiling,^5^ identifying biomarkers to predict toxicity to cancer treatment has been limited.^6^ Notably, toxicity to immune therapy occurs in 25%–30% of patients regardless of cancer type, even in non-responding tumor types, supporting the hypothesis that toxicity is patient-specific and not tumor type-dependent. This further indicates that there are tumor-specific factors predicting response to immune therapy, which has been demonstrated, as described above. However, we hypothesized that since toxicity likely represents a patient-specific systemic immune stress response, biomarkers of toxicity should be found in a patient’s germline DNA. Unfortunately, predictive germline variants in the most studied regions of the DNA—the protein coding regions—are quite rare,^7^ and approaches that have been historically applied to study the ‘non-coding’ regions of the DNA, such as genome-wide association studies (GWAS), have been relatively unsuccessful in identifying functional germline variants.

Fortunately, the ability to identify meaningful biomarkers in the non-coding germline DNA has been dramatically advanced with the discovery of functional non-coding regions,^8^ such as those encoding microRNAs (miRNAs) and their regulatory pathways.^9^ miRNAs are global gene regulators that play important roles in cancer^10^ and are critical regulators of the systemic stress response, including the immune response to cancer therapy.^11–13^ Recent work has identified functional variants in miRNA pathways (now often referred to as miRNA single nucleotide polymorphisms or miRSNPs), which have dramatic impacts on response to cancer therapy.^14 15^ Notably, to date, miRSNPs are poorly represented in widely used single nucleotide polymorphism (SNP) platforms as well as in exon sequencing. The first example of a cancer-associated miRSNP is a functional miRNA binding site variant in the 3 prime untranslated region (3′UTR) of the *KRAS* oncogene,^16^ now referred to as the *KRAS*-variant, which is a biomarker that predicts significant differences in treatment response or resistance in a pan-cancer manner.^17–21^ Furthermore, it was shown that *KRAS*-variant patients are immunosuppressed with significantly elevated transforming growth factor beta (TGF-ß), higher toxicity, and a poor systemic response to radiation.^22^ In addition, it was recently found that a panel of miRSNPs predict wound toxicity after accelerated radiation in sarcoma.^23^ These findings afford evidence of the global impact this class of functional variants can have on the systemic stress response, resulting in toxicity to cancer therapies.

We therefore evaluated a panel of miRSNPs (previously discovered by our group)^24^ in patients treated with anti-programmed death 1 (anti-PD1)/programmed death-ligand 1 (PDL1) checkpoint therapy to determine if they can act as biomarkers of increased risk for irAEs, regardless of cancer type.

## Materials and methods

### Patients

Based on sample availability, the study included 161 patients with cancer treated with single-agent anti-PD1 or anti-PDL1 therapy. All were consented on one of three Human Investigations Committee-approved protocols: ClinicalTrials.gov number NCT01295827 (IRB#11-003066, initiated in 2011), MiraKind protocol Pro00009633 (initiated in 2015), or ClinicalTrials.gov number NCT02280161 (IRB#14-001115, initiated in 2014). All samples were processed and tested through MiraDx on their miRSNP platform. Cycle number, toxicity status, and in some cases tumor responses were collected directly from patient charts by physicians with protocol access rights. Inclusion criteria for this study were the following: having received equal to or more than four cycles of anti-PD1/PDL1 therapy (unless stopping due to toxicity before cycle 4); having therapy delivered every 2 or 3 weeks; not being on steroids for other reasons; and not having a pre-existing diagnosis of an autoimmune disease prior to treatment.

irAEs were recorded by the treating physician and graded per American Society of Clinical Oncology guidelines as grades 1–4.^25^ irAEs were considered significant if they were recorded as grade 2 or higher and the patient required a treatment break and steroid therapy for resolution. The choice of grade 2 or higher as the cut-off for significance was based on prior published toxicity studies,^26^ the rarity of grade 3 and higher toxicity to single-agent anti-PD1/PDL1, and the clarity of this toxicity through the need for steroids and a treatment interruption. Response was recorded by treating physicians for the melanoma clinical trial patients using immune-Response Evaluation Criteria in Solid Tumors (iRECIST) criteria,^27^ using best overall response as follows: progressive disease (PD), stable disease (SD), partial response (PR), or complete response (CR).

Because the markers tested are germline and the study was a retrospective analysis of prospective studies, the samples studied were collected at any point in treatment. Patients with melanoma were primarily treated on pembrolizumab or nivolumab protocols at University of California, Los Angeles (UCLA) for advanced and relapsed disease, with a small proportion treated with anti-PDL1 agents. Patients with prostate cancer all had metastatic prostate cancer and were treated with anti-PD1 single-agent pembrolizumab therapy at Prostate Oncology Specialists and consented to a MiraKind protocol. Patients with other cancer types were captured through the radiation oncology or urology clinics at UCLA or were collected at Saint John’s Cancer Center or Appalachian Regional Healthcare and submitted to MiraDx directly for analysis. These patients were a mix of metastatic patients and patients with earlier-stage, definitively treated NSCLC or GU cancer. All were treated with anti-PD1 or anti-PDL1 agents, including pembrolizumab, nivolumab, atezolizumab, or avelumab. The proportion of patients treated with anti-PD1 versus anti-PDL1 therapy by cancer type is presented in online supplemental table 1.

10.1136/jitc-2021-003625.supp1 Supplementary data

### DNA biomarker panels

DNA was isolated from blood or saliva using Qiagen kits per protocol. Because prior reports have not shown any differences in the prevalence of germline variants in blood versus saliva, either was used as source of germline DNA samples.^16^ Biomarkers were chosen from a pool of miRNA-based variants disrupting DNA repair and immune-related genes previously discovered and determined to be functional through sequencing and bioinformatic approaches.^24^ MiraDx performed the miRSNP analysis on blinded samples. Additional information on variant selection for testing and comparison between training and validation cohorts are included in online supplemental methods, part 1 and online supplemental table 2.

### Statistical analysis

Four classifiers were trained on the largest disease-homogenous cohort, a set of 62 patients with melanoma with documented toxicity. Subjects were classified as experiencing high toxicity (grade 2 or higher) versus low toxicity (lower than grade 2). Performance of the classifiers was tested on a validation set of 99 patients with other types of cancer. Classifiers were built using classification trees (CT),^28^ least absolute shrinkage and selection operator (LASSO)-regularized logistic regression (LASSO-LR),^29^ boosted trees (BT),^30^ and random forests (RF).^31^ Due to the class imbalance in observed toxicity, subjects with toxicity were upweighted in the training data and tuning parameters were selected to minimize the F1 score (defined as the harmonic mean of sensitivity and positive predictive value) using leave-one-out cross validation (LOOCV). The final performance measures, accuracy, specificity, sensitivity, negative predictive value, positive predictive value, area under the curve (AUC), and F1 score were reported on both the training data using LOOCV and on the validation cohorts using data held out from model training. A total of four missing marker values were imputed five times via chained equations,^32^ with markers treated as categorical variables. Results are presented for a single imputation data set given that performance and variable importance measures are stable across imputation data sets. Additional information on statistical analyses is included in online supplemental methods, part 2.

## Results

### Toxicity across patient cohorts

For the melanoma training cohort, 21 of 62 patients (33.9%) developed grade 2 or higher irAEs, and for the validation cohort 24 of 99 patients (24.2%; 25% of prostate and 23.8% of other) developed grade 2 or higher irAEs. The frequency and distribution of toxicity did not vary significantly by cancer type (p=0.425; online supplemental table 3), even in cancer types without expected response to immune therapy. The patients in the validation cohort had several different types of cancer, including prostate, non-prostate GU cancer, NSCLC, head and neck cancer, sarcoma, and others. Forms of grade 2 or greater toxicity, in order of frequency, included skin, colon, thyroid, adrenal, liver, lung, muscular, arthritis, neurologic, renal, fatigue, and cardiac (table 1).

**Table 1:** Description of patients across cancer types

	Toxicity grade <2 (n=116)	Toxicity grade ≥2 (n=45)	Overall (N=161)	P value
Total cycles, median (IQR)	9 (12)	15 (18)	10 (15)	0.229
Toxicity cycle, median (IQR)	–	8 (16)	–	
Male, % (n)	69 (80)	78 (35)	71 (115)	0.322
Age, mean (SD)	66 (14)	67 (11)	67 (13)	0.691
Cancer type, % (n)				0.425
Melanoma	35 (41)	47 (21)	39 (62)	
Prostate	23 (27)	20 (9)	22 (36)	
Other	41 (48)	33 (15)	39 (63)	
HNSCC	5 (6)	0 (0)	4 (6)	
GU	9 (11)	18 (8)	12 (19)	
GYN	3 (4)	2 (1)	3 (5)	
NSCLC	9 (11)	13 (6)	11 (17)	
Miscellaneous (sarcoma, GYN, breast, GBM, GI)	14 (16)	0 (0)	10 (16)	
Toxicity type, % (n)				
Adrenal	–	13 (6)	–	
Arthritis	–	4 (2)	–	
Skin	–	36 (16)	–	
Colitis	–	18 (8)	–	
Fatigue	–	2 (1)	–	
Muscular/skeletal	–	7 (3)	–	
Kidney	–	2 (1)	–	
Liver	–	13 (6)	–	
Lung	–	9 (4)	–	
Neurologic	–	4 (2)	–	
Thyroid	–	16 (7)	–	
Cardiac	–	2 (1)	–	

The reported p values are associated with independent z tests for difference in mean for continuous variables, z tests for differences in proportions for counting variables, and χ^2^ tests for categorical variables.

GBM, glioblastoma multiforme; GI, gastrointestinal; GU, genitourinary cancer; GYN, gynecological; HNSCC, head and neck cancer; NSCLC, non-small cell lung cancer.

**Figure 1:**
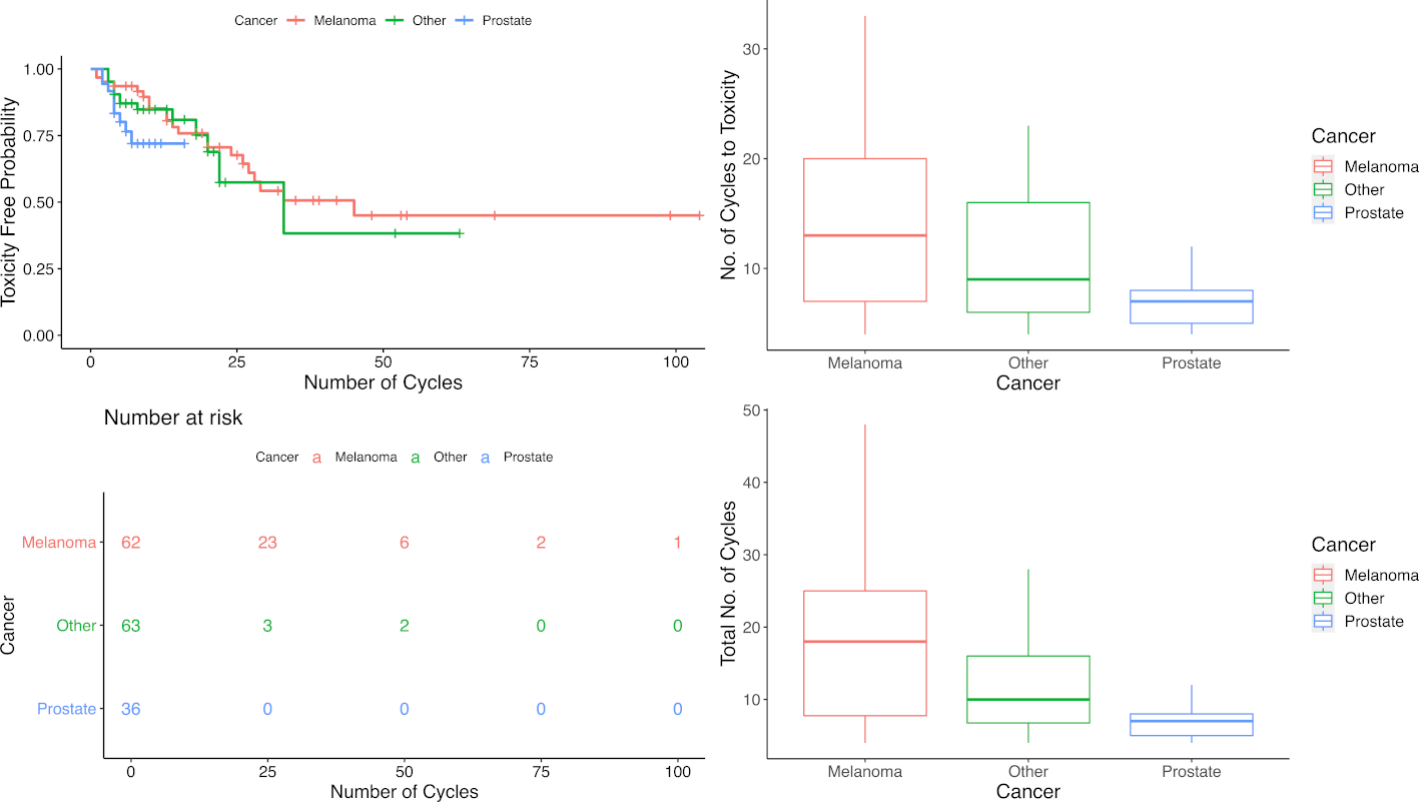
Toxicity-free probability survival curves and cycles across cancer types. (Top left) Survival curves of toxicity-free probability (grade 2 or higher) stratified by cancer type by number of cycles. (Bottom left) Risk table for toxicity-free survival (grade 2 or higher) stratified by cancer type by number of cycles. (Top right) Box plot of number of cycles to toxicity by cancer type. (Bottom right) Box plot of total number of cycles by cancer type.

For analysis the validation cohort was grouped as prostate and other. The probability of experiencing grade 2 or higher irAEs in all groups increased with increasing cycles of anti-PD1/PDL1 therapy delivered. As far as the onset of toxicity, patients with melanoma had the longest time before developing toxicity (median number of cycles=14, IQR=17) and patients with prostate cancer had the shortest time before developing toxicity (median number of cycles=4, IQR=2), with the three cancer groups (melanoma, prostate, and other) differing only marginally in their estimated toxicity-free survival time (log-rank test, p=0.110; figure 1).

### Models and biomarkers predicting toxicity

Within the melanoma training cohort, toxicity was predicted with 79.4% accuracy for CT (F1=0.748, AUC=0.753), 80.3% accuracy for LASSO-LR (F1=0.750, AUC=0.816), 82.0% accuracy for BT (F1=0.744, AUC=0.806), and 75.4% accuracy for RF (F1=0.681, AUC=0.756). The consistency of findings across these different statistical approaches suggests strong predictive performance is not restricted to a particular class of models.

The same classifiers were then applied to the validation cohort to determine their ability to predict toxicity regardless of cancer type on held-out samples. The same biomarkers predicting toxicity for patients with melanoma predicted grade 2 or greater irAEs in the validation set, with 77.0% accuracy for CT (F1=0.593, AUC=0.786), 77.6% accuracy for LASSO-LR (F1=0.621, AUC=0.788), 79.6% accuracy for BT (F1=0.571, AUC=0.724), and 79.6% accuracy for RF (F1=0.600, AUC=0.746). The accuracy was better in the ‘other cancer types’ versus the prostate cancer cohort, perhaps due to the shortest duration of treatment resulting in the lowest positive predictive value for these patients. Yet, overall, the classifiers performed consistently very well in both validation cohorts. The receiver operating characteristic curve (ROC) shows the trade-off between sensitivity and specificity at various decision thresholds for LASSO-LR for each of the cancer subgroups (online supplemental figure 1). For the melanoma and validation groups, the ROC curve lies above the 45° diagonal, with an average AUC of 0.797, showing the strong performance of the LASSO-LR classifier. The ability for the final biomarker signature to predict toxicity is shown for the four tuned classifiers applied from a single imputation (table 2). The final cross-validated tuning parameters for the four classifiers are presented in online supplemental methods, part 3.

**Table 2:** Performance measures for the four classifiers

	Accuracy	Sensitivity	Specificity	PPV	NPV	F1	AUC
Training: melanoma (LOOCV estimate)							
Classification trees	0.794	0.840	0.774	0.667	0.914	0.753	0.748
LASSO-LR	0.803	0.905	0.750	0.655	0.938	0.760	0.827
Random forest	0.754	0.762	0.750	0.615	0.857	0.681	0.756
Boosted trees	0.820	0.762	0.850	0.727	0.872	0.744	0.806
Test: prostate and other cancers							
Classification trees	0.770	0.660	0.809	0.553	0.884	0.593	0.786
LASSSO-LR	0.776	0.773	0.783	0.514	0.921	0.621	0.778
Random forest	0.796	0.840	0.652	0.556	0.887	0.600	0.746
Boosted trees	0.786	0.840	0.609	0.538	0.875	0.571	0.724
Test: prostate cancer							
Classification trees	0.622	0.550	0.661	0.353	0.818	0.435	0.648
LASSO-LR	0.667	0.667	0.667	0.400	0.857	0.500	0.667
Random forest	0.750	0.815	0.556	0.500	0.846	0.526	0.685
Boosted trees	0.750	0.778	0.667	0.500	0.875	0.571	0.722
Test: other cancers							
Classification trees	0.844	0.719	0.888	0.687	0.915	0.710	0.869
LASSO-LR	0.839	0.833	0.857	0.600	0.952	0.706	0.845
Random forest	0.839	0.854	0.786	0.611	0.932	0.688	0.820
Boosted trees	0.806	0.875	0.571	0.571	0.875	0.571	0.723

Results are reported for the melanoma training data (best LOOCV estimate) and the validation data.

AUC, area under the curve; F1, F1 score; LASSO-LR, LASSO-regularized logistic regression; LOOCV, leave-one-out cross validation; NPV, negative predictive value; PPV, positive predictive value.

**Figure 2:**
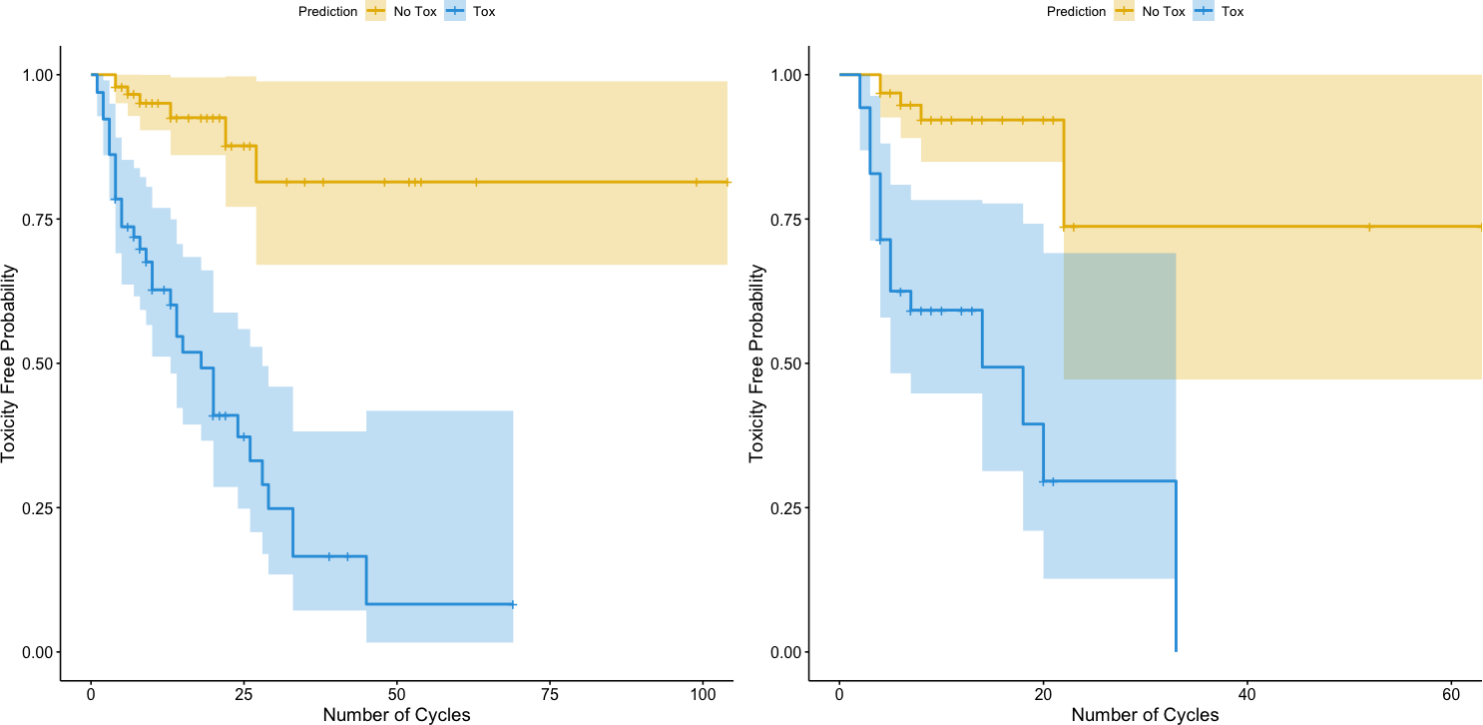
Survival curves of toxicity-free probability stratified by our biomarker signature. The orange lines are the estimated toxicity-free probability survival curves for patients who were predicted to not experience toxicity (probability of toxicity <0.5), while the blue lines are the estimated survival curves for patients who were predicted to have toxicity (probability of toxicity ≥0.5). The left panel includes the entire cohort and all cancer types. The right panel includes only the test set. HR estimated through a Cox proportional hazards and p value estimates via log-rank tests.

Our toxicity biomarker signature conferred a substantially increased risk of toxicity with increasing cycles of treatment, with a greater than ninefold increase in toxicity for these patients for the entire cohort (figure 2, left panel) as well as for patients in the test set alone (figure 2, right panel) (p<0.001) compared with patients for whom our biomarker signature did not predict toxicity. This finding is also found when dividing the test set into prostate only versus others (online supplemental figure 2). These findings suggest that patients with this toxicity biomarker signature eventually develop toxicity over time with increasing exposure to treatment, seemingly regardless of cancer type.

To characterize the biomarkers that were the most predictive of toxicity, marginal associations and variable importance measures were calculated. Marginally, a 3′UTR germline mutation in *RAC1* (rs9374) was identified as the most significant biomarker in predicting toxicity across cancers and within both the melanoma and other cancer subgroups (online supplemental table 4). Similarly, *RAC1* was identified as the most influential marker in all four classifiers by a factor of at least two in their respective variable importance measures (online supplemental figure 3).

### The association of biomarkers of toxicity with response to checkpoint therapy

We next investigated if our identified germline biomarkers predicting toxicity would also predict response to anti-PD1/PDL1 therapy. Within the melanoma cohort, we evaluated the association between predicted probabilities of toxicity using LASSO-LR and response to anti-PD1/PDL1 therapy. Response was documented as CR, PR, SD, or PD per protocol. Kendall’s rank correlation between probability of toxicity and response category was estimated at −0.04 (p=0.71); thus, there was no apparent association.

In the same cohort, we also evaluated the association between response and the single strongest biomarker of toxicity in *RAC1*. We found that, overall, heterozygous *RAC1* status was not associated with any category of response in these patients using χ^2^ test of independence (p*=*0.151; online supplemental table 5). Finally, we evaluated if there was an association between irAE grade and any category of response in our melanoma cohort, but also did not find a clear association (p=0.539; online supplemental table 6).

## Discussion

In this study we identify a discrete germline toxicity biomarker panel that predicts grade 2 or higher irAEs to single-agent anti-PD1/PDL1 checkpoint therapy, with an accuracy of over 77%. This panel consists of inherited, germline mutations primarily in regions disrupting miRNAs, referred to as miRSNPs. These regions are logical locations for such biomarkers, as miRNAs themselves are known to be first responders in the systemic stress response.^11^ Furthermore, consistent with the clinical evidence that irAEs are a patient-specific and pan-cancer phenomenon, our biomarker panel predicts irAEs across, or regardless, of cancer type. Finally, and perhaps not surprisingly, our genetic toxicity panel alone did not appear to predict a patient’s response to single-agent anti-PD1/PDL1 therapy.

Other approaches to try and identify patients at risk of irAEs have used baseline gene profiling in whole blood^33^ or circulating cytokines before and after the initiation of treatment.^26 34^ The baseline gene profiling approach found some evidence for baseline biomarkers and is consistent with the hypothesis that irAEs are patient-specific, but this approach has not been applied to anti-PD1/PDL1 therapies nor has it been investigated across cancer types. The circulating cytokine work found that baseline interleukin (IL) 17 or on-treatment IL-17 levels could predict irAEs with ipilimumab in melanoma alone, but this has not been further validated. The most recent study, which was the largest, looked at circulating cytokines before and after treatment initiation for patients with melanoma treated with anti-PD1 or anti-cytotoxic T-lymphocyte-associated antigen 4 (CTLA-4) plus anti-PD1 therapy.^26^ While the study did not find cytokines predicting toxicity to single-agent anti-PD1 therapy, they did find cytokines predicting toxicity to combination anti-PD1 plus anti-CTLA-4 therapy, with an AUC of 0.78 in their training cohort (n=58) and an AUC of 0.68 in their validation cohort (n=49). Notably, like our findings, they did not find an association between response and any of the cytokines being studied or an association of response with their toxicity score.

Although a potential weakness of our study is the relatively small number of individuals studied, we believe that this is balanced by many strengths of our study, one of which is the use of several cohorts of patients from different institutions in which we were able to validate our biomarker panel. This is in fact the largest cohort reported of patients studied for germline biomarkers of irAEs, and its validation in independent cohorts further confirms its significance. It is important to note that the patients in these studies also had diverse prior treatments, with some receiving adjuvant treatment after surgery and others joining the original Keynote trials where the anti-PD1/PDL1 treatment was their final option. Our findings that this biomarker signature predicts toxicity regardless of prior treatment support the hypothesis that these biomarkers are related to a fundamental inherent systemic response, versus tumor mutations or the tumor milieu, which has been shown to evolve with prior therapy.

While the specific 3′UTR variant in *RAC1* identified in our study has not been broadly studied, the *RAC1* protein itself is important in both innate immunity^35^ as well as autoimmunity,^36 37^ supporting our findings that dysregulation of this gene could be reasonably expected to be predictive of irAEs. In addition, another important biomarker identified as having high variable importance across our models is the IL-10 receptor 2 SNP rs2834167. This variant has been associated with an increased susceptibility to systemic sclerosis,^38^ and its involvement in our signature is consistent with the known increased risk of irAEs in patients with known pre-existing autoimmunity.^39^ It is also pertinent to note that others have found that the level of the IL-10 cytokine predicts response to anti-CTLA-4 therapy, supporting the importance of this signaling pathway in immune response.^34^

Another interesting finding in this work is the lack of an apparent association between our signature or most predictive germline biomarker of toxicity in *RAC1* and response to anti-PD1/PDL1 checkpoint therapy. It is important to acknowledge that this study is not adequately powered to detect patterns of association between toxicities and response rates, especially if that association exists primarily between higher-grade irAEs and response, due to the expected rarity of grade 3 and above irAEs from anti-PD1/PDL1 single-agent therapy as seen in our study. In addition, even in the presence of a mild positive association between irAE grade and response, germline signatures predicting toxicity are unlikely to predict response with an acceptable degree of accuracy. However, we would also hypothesize that germline signatures, at least alone, are unlikely to be able to predict response, as response to checkpoint therapy is very tumor type-dependent, suggesting a strong impact of tumor-specific factors. As larger sample numbers are accrued, our approach will be extended to include both germline factors and tumor biomarkers to better define the role, if any, of germline biomarkers in predicting response to checkpoint therapy, work that is ongoing.

The ability to predict toxicity to checkpoint therapy before the initiation of treatment has broad potential clinical utility. For example, checkpoint inhibitors are now being applied in the adjuvant setting where individual benefit is harder to ascertain. In addition, checkpoint therapy is being broadly offered in the metastatic cancer setting as a palliative therapy. The likelihood of serious toxicity could prove to be a critical factor in deciding whether to implement therapy in such settings or it could lead to altered management through cycle reductions or closer surveillance if the potential benefit outweighs the risk of treatment. Importantly, these findings should also be taken into account when considering the increasingly important concept of financial toxicity to patients with cancer. Finally, anti-PD1/PDL1 drug therapy is often considered a safe base to which additional immune-stimulating agents can be added, where the risk of more severe toxicity is high. Work to determine if our germline signature of toxicity to single-agent anti-PD1/PDL1 is predictive of increased toxicity risk to combination therapy is ongoing.

While we are still at the early stages of understanding the mechanisms by which miRNA-based germline mutations regulate immunity and the systemic stress response, it is important to note that our repeated findings that germline variant panels can reproducibly predict systemic toxic responses to cancer therapy are potentially paradigm-shifting. Application of this class of functional variants may improve our ability to offer truly personalized cancer therapy by enabling consideration of toxicity along with patient response. As the efficacy of cancer therapy improves, resulting in higher and higher rates of long-term cancer control, cure without harm will only become an increasingly important endpoint.

## Data Availability

Data are available upon reasonable request.
