# Supplementary material for: Germline biomarkers predict toxicity to anti-PD1/PDL1 checkpoint therapy
Source: J Immunother Cancer. 2022 Jan 31;10(2):e003625. doi: 10.1136/jitc-2021-003625 (PMC8804679; doi:10.1136/jitc-2021-003625)
Supplement: Supplementary data [file jitc-2021-003625supp001.pdf]

Supplemental Data

Supplemental Methods:

Part 1: We hand-checked every single variant on our final list using RNA Hybrid, a software program that anneals RNA sequences in trans.[26] 3'UTR variants were assessed for their ability to (negatively or positively) disrupt miRNA binding according to the 'old' and 'new rules' for miRNA-target site recognition.[27-31] As a final step in our evaluation for candidate variants to test, we limited ourselves to variants predicted to be found in between 0.5% to 50% of the population, as we were looking for biomarkers that are likely to be detected in reasonably small cohorts of patients. Our search initially filtered out 2,540 total variants, which we reduced to a final list of ~350 variants by fitting the above defined priority parameters for miRNA pathway variants, and included an additional ~150 variants in coding sequences. Of note, many of the variants included in this analysis are not included in the most recent Illumina or GWAS platforms. Panels were run using the Sequenome platform, and analysis included approximately 500 single nucleotide polymorphisms or deletions. Each panel was run with internal controls that used Taqman Genotyping as the gold standard. Any biomarker with less than a 90% call rate or more than 1% error found by controls was excluded from further analysis. For our final 500 biomarkers, to insure sufficient marginal variation in the final panel, any biomarker with an observed rate of mutation less than 5.0% in the training sample was excluded from the analysis. The final statistical analysis included 50 common biomarkers between training and validation samples (**Supplemental Table 1**).

Weidhaas J, *et al. J Immunother Cancer* 2022; 10:e003625. doi: 10.1136/jitc-2021-003625

Part 2: CT were tuned separately on minimum split and minimum node size, LASSO-LR models were tuned on the regularization parameter lambda, BT were tuned on the learning parameter eta, tree depth, and the number of rounds, and RF were tuned on number of trees and variables considered at each split. CT, LASSO-LR, BT, and RF classifiers were fit in R (version 3.5.1)[36] with mlr (version 2.1.1) calling glmnet (version 2.0-10), xgboost (version 0.71.2), and ranger (version 0.10.1), respectively. Imputation was performed in R calling mi (version 1.0) with a maximum of 20 iterations. Marginal tests of association were performed using stats (version 3.5.1) to compare total cycles, gender, age, cancer grouping, and all biomarkers between the toxic and non-toxic groups. For continuous variables, Wilcoxon rank-sum test and Welch's t-tests were performed for non-normal and normal data, respectively.[38] For categorical variables, chi-square tests of independence were performed with p values calculated by Monte Carlo tests.[39] For marginal tests of association of biomarkers in each cancer group (all, melanoma, prostate, and other), raw p values were reported along with their corresponding q values[40] using qvalue (version 2.10.1). Significant differences among survival functions were assessed using the log-rank test[41] performed with survival (version 2.42-6). Differences between cancer types measuring the onset of toxicity in terms of number of treatment cycles were assessed via a Kruskal-Wallis rank-sum test. Variable importance measures were obtained for each of the tuned classifiers. Importance was calculated for CT using the cumulative decrease in Gini impurity for primary and surrogate splits at each node, for LASSO-LR as the absolute value of regression weights, for BT as the relative information gain of each features across all nodes, and for RF as the mean decrease in the Gini impurity across all splits. Importance measures were rescaled to sum to one to facilitate comparisons across classifiers.

All plots were generated using ggplot2 (version 3.0.0). Receiver operating characteristic (ROC) curves were calculated using plotROC (version 2.2.1) and survival curves were calculated using survminer (version 0.4.3) calling survival (version 2.42-6).

Part 3: Cross validated tuning parameter values

The final cross validated tuning parameters for the reported classifiers are as follows: minimum node size 5 for classification trees (CT), regularization parameter lambda equal to exp(-2.5) for LASSO-regularized logistic regression (LASSO-LR), learning parameter eta of 0.1, max depth of 5, and 5 rounds for boosted trees (BT), and ten variables considered at each split with 200 trees for random forests (RF). For each classifier, all remaining hyperparameters were assigned their default values as defined through their associated R packages.

Weidhaas J, *et al. J Immunother Cancer* 2022; 10:e003625. doi: 10.1136/jitc-2021-003625

***Supplemental Table 1**: Anti-PD1 and Anti-PDL1 prevalence in three cancer cohorts.*

	Anti-PD1 No. (%)	Anti-PDL1 No. (%)
Melanoma	61 (98.4%)	1 (1.6%)
Other	51 (81.0%)	12 (19.0%)
Prostate	36 (100%)	0 (0.0%)

***Supplemental Table 2**: 50 common markers between training and validation data sets. Percent values report Hardy-Weinberg minor allele frequencies (MAF) in our sample. Reported MAF from dbSNP are also listed.*

Name	MAF	MAF dbSNP	Name	MAF	MAF dbSNP
IL10RB_rs2834167	0.31	0.32	HAMP_rs10421768	0.20	0.20
RAC1_rs9374	0.25	0.19	IL10_rs3024496	0.44	0.42
ABL1_rs11991	0.09	0.18	IL12A_rs568408	0.11	0.14
ATM_rs1801516	0.14	0.11	IL18R1_rs1146566	0.09	0.11
ATM_rs189037	0.46	0.48	IL1A_rs1800587	0.30	0.31
BMP2_rs235768	0.31	0.33	IL2RA_rs11256497	0.39	0.27
BRCA1_rs8176318	0.29	0.29	IL6_rs1800795	0.35	0.32
BRCA2_rs15869	0.19	0.16	IL8_rs4073	0.49	0.46
BRCA2_rs7334543	0.25	0.26	LIG4_rs3093772	0.12	0.10
KIT_rs17084733	0.09	0.09	LIN28A_rs9438623	0.12	0.13
CAMK2G_rs2306327	0.14	0.14	MDM2_rs769412	0.05	0.05
CD274_rs1411262	0.26	0.33	miR-34b/c_rs4938723	0.36	0.33
CD274_rs2282055	0.24	0.25	miR-99a promoter	0.38	novel
CD274_rs2297136	0.41	0.42	MSH2_rs2303428	0.11	0.12
CD274_rs4143815	0.31	0.28	NBN_rs1805794	0.34	0.35
CD274_rs4742098	0.26	0.26	P2RX7_rs3751143	0.19	0.19
CD274_rs822339	0.25	0.24	REV3L_rs465646	0.20	0.19
CD6_rs76677607	0.07	0.05	KRAS_rs61764370	0.12	0.06
CSMD1_rs583087	0.09	0.11	RAD23A_rs8240	0.08	0.04

Weidhaas J, *et al. J Immunother Cancer* 2022; 10:e003625. doi: 10.1136/jitc-2021-003625

EGFR_rs884225	0.09	0.10	SMAD1_rs11724777	0.39	0.32
ERCC1_rs3212948	0.37	0.34	STAT3_rs3744483	0.23	0.26
ERCC4_rs4781562	0.24	0.26	TNNT2_rs3729843	0.42	0.33
EREG_rs1460008	0.19	0.19	TP53INP1_rs7760	0.11	0.18
EXO1_rs4150021	0.15	0.18	XRCC2_rs3218536	0.07	0.06
FCGR2A_rs1801274	0.48	0.48	XRCC3_rs861539	0.33	0.31

Supplemental Table 3*: irAE Grade distribution by cancer cohort*

	Melanoma No. (%)	Prostate No. (%)	Other No. (%)
irAE Grade 0	18 (29.0%)	16 (44.4%)	23 (36.5%)
irAE Grade 1	23 (37.1%)	11 (30.6%)	25 (39.7%)
irAE Grade 2	17 (27.4%)	3 (8.3%)	15 (23.8%)
irAE Grade 3	3 (4.8%)	4 (11.1%)	0 (0%)
irAE Grade 4	1 (1.6%)	2 (5.6%)	0 (0%)

Supplemental Table 4: *Marginal associations among markers (0 = WT, 1 = heterozygous mutant, 2 = homozygous mutant) and no toxicity (NT) and toxicity (T) across and within cancer types. Variants with marginal p values less than .010 are displayed for each category.*

**Variants**	**NT: 0**	**NT: 1**	**NT: 2**	**T: 0**	**T: 1**	**T: 2**	***p* value**	***q* value**
**<u>All cancers</u>**								
RAC1_rs9374	82	27	7	6	38	1	<.001	<.001
ATM_rs1801516	93	21	2	27	15	3	0.016	0.400
EGFR_rs884225	92	22	2	43	2	0	0.04	0.533
CD274_rs2297136	44	54	18	17	14	14	0.060	0.533
FCGR2A_rs1801274	33	50	33	6	28	11	0.060	0.533
**<u>Melanoma</u>**								
RAC1_rs9374	27	11	3	2	19	0	<.001	<.001

miR-99a promoter	9	21	11	12	7	2	0.014	0.35
ERCC1_rs3212948	18	22	1	5	12	4	0.028	0.467
EGFR_rs884225	34	7	0	21	0	0	0.082	0.867
SMAD1_rs11724777	14	24	3	5	10	6	0.096	0.867
**Prostate**								
ATM_rs1801516	24	3	0	5	4	0	0.046	1.00
IL10_rs3024496	9	10	8	2	7	0	0.053	1.00
**Other**								
RAC1_rs9374	38	7	3	2	13	0	<.001	<.001
XRCC3_rs861539	20	21	7	11	4	0	0.066	1.00
FCGR2A_rs1801274	14	23	11	1	12	2	0.077	1.00

Supplemental Table 5*: Response to anti-PDL-1/PDL-1 therapy stratified by RAC-1 among melanoma patients. RAC-1 categories are collapsed to match the learned toxicity signature. Response is divided into four categories: Progressive Disease (PD), Stable Disease (SD), Partial Response (PR) and Complete Response (CR). RAC_1=1 is the heterozygous patients, RAC_1 = 0 homozygous wild-type, and RAC_1 = 2 homozygous variant.*

	**RAC_1 = 0,2**	**RAC_1 = 1**	***p* value**
**CR**	8	2	0.151
**PR**	11	14	
**SD**	2	5	

Weidhaas J, *et al. J Immunother Cancer* 2022; 10:e003625. doi: 10.1136/jitc-2021-003625

**PD**	11	8	

Supplemental Table 6: irAE Grade distribution vs. response rates in the melanoma cohort. *In the table no response reflects SD and PD, and response reflects PR and CR. We do not see a significant association in this small cohort (p=0.539).*

	**No Response no. (%)**	**Response no. (%)**
**irAE Grade 0**	10 (55.6%)	8 (44.4%)
**irAE Grade 1**	10 (43.5%)	13 (56.5%)
**irAE Grade 2**	6 (35.3%)	11 (64.7%)
**irAE Grade 3**	0 (0.0%)	2 (100.0%)
**irAE Grade 4**	0 (0.0%)	1 (100.0%)

Weidhaas J, *et al. J Immunother Cancer* 2022; 10:e003625. doi: 10.1136/jitc-2021-003625

***Supplemental Figure 1**: ROC curve for LASSO-Logistic Regression from a single imputation. The ROC within the melanoma cohort is obtained via LOOVC. The prostate and other cohorts are treated as a test set.*

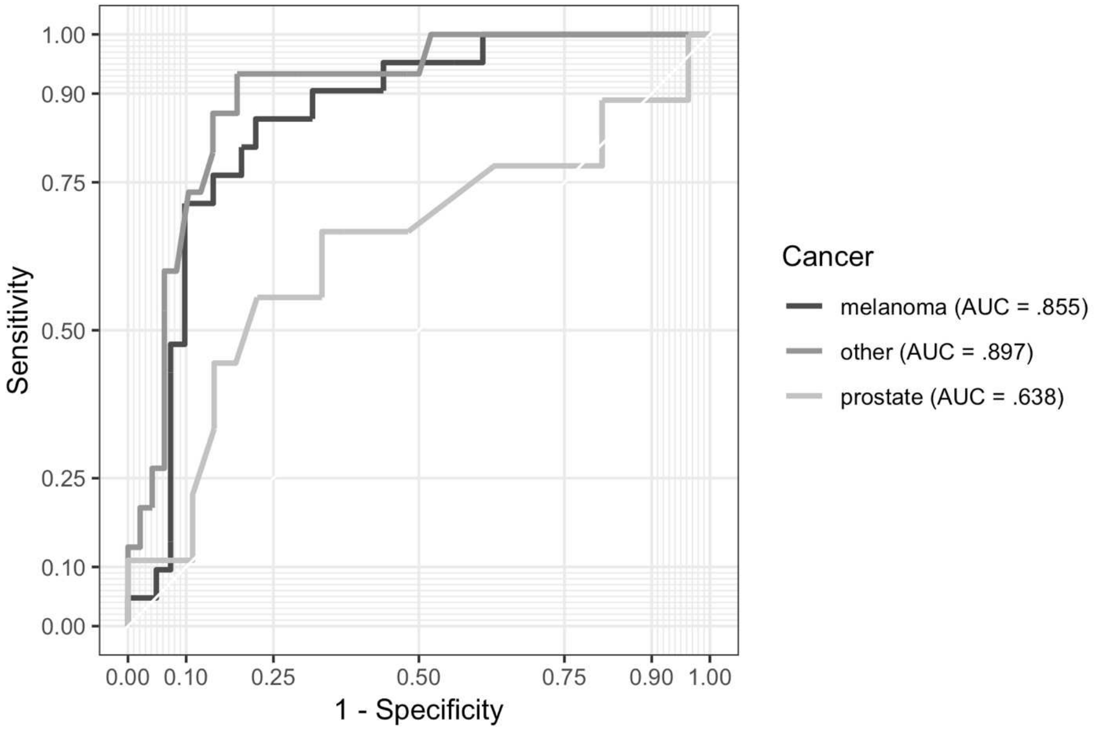

Weidhaas J, *et al. J Immunother Cancer* 2022; 10:e003625. doi: 10.1136/jitc-2021-003625

Supplemental Figure 2**: **Survival curves of toxicity free probability for the test sets stratified by our biomarker signature. Orange lines are the estimated toxicity free probability survival curves for patients that were predicted to not experience toxicity (probability of toxicity < 0.5), while the blue lines are the estimated survival curves for patients that were predicted to have toxicity (probability of toxicity >= 0.5). Left panel includes prostate only. Right panel includes other cancer types. Hazard ratios (HR) estimated through a cox proportional hazards, and p value estimates via log-rank tests.

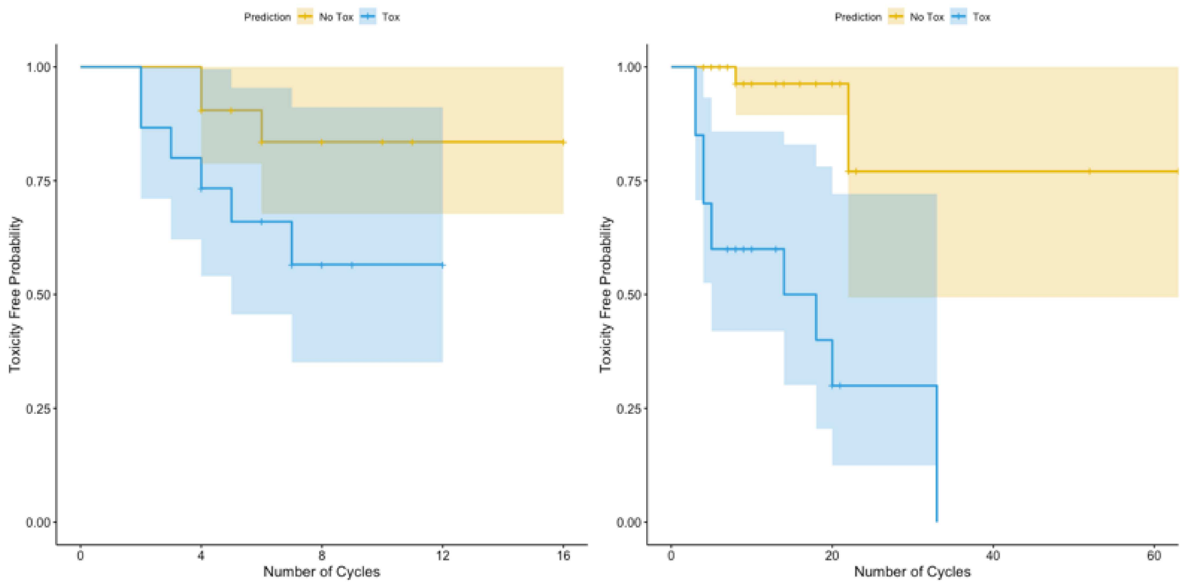

Weidhaas J, *et al. J Immunother Cancer* 2022; 10:e003625. doi: 10.1136/jitc-2021-003625

***Supplemental Figure 3**: Importance of top markers by classifier.*

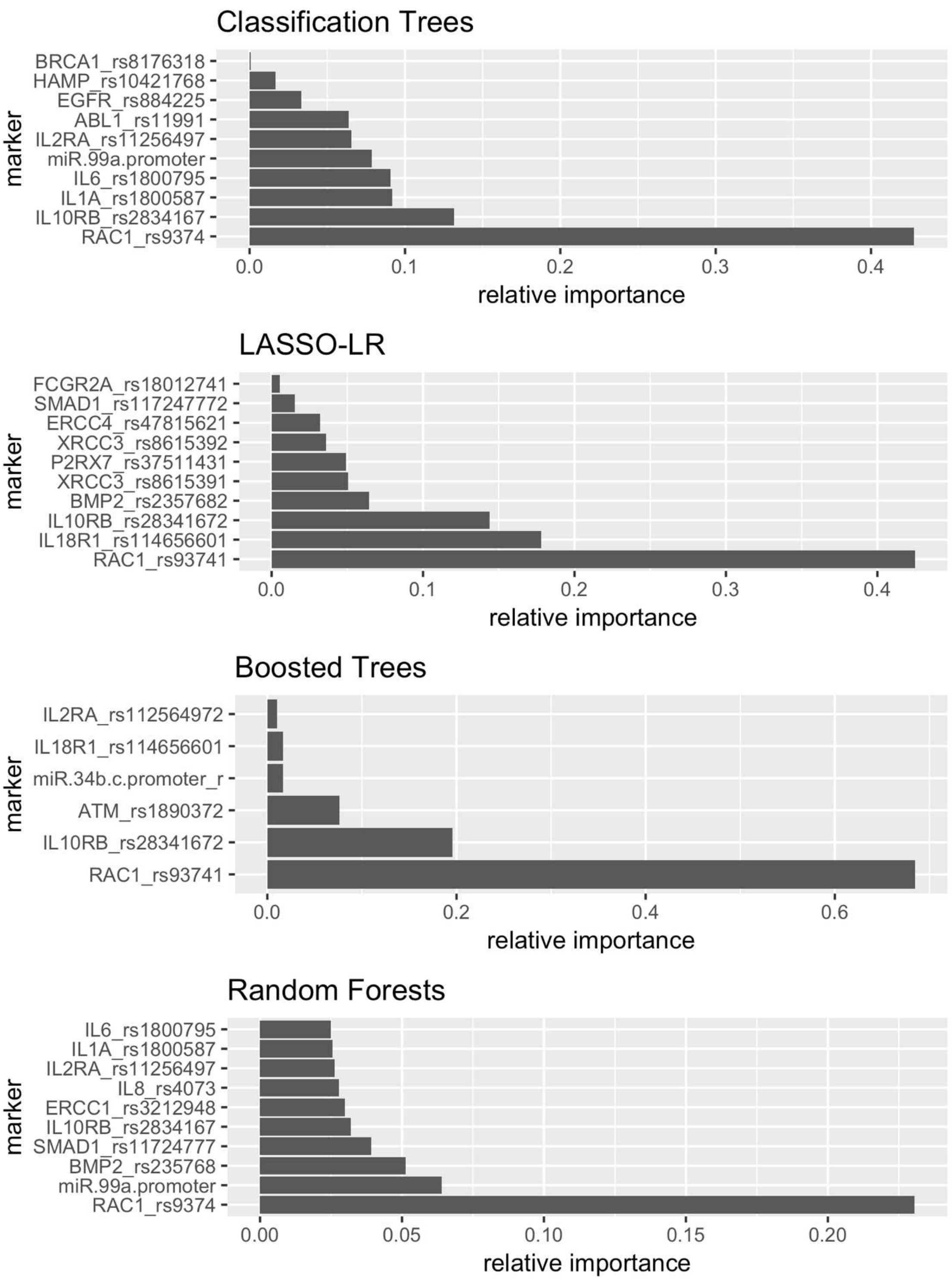
Classification Trees
marker
BRCA1_rs8176318
HAMP_rs10421768
EGFR_rs884225
ABL1_rs11991
IL2RA_rs11256497
miR.99a.promoter
IL6_rs1800795
IL1A_rs1800587
IL10RB_rs2834167
RAC1_rs9374
0.0
0.1
0.2
0.3
0.4
relative importance
LASSO-LR
marker
FCGR2A_rs18012741
SMAD1_rs117247772
ERCC4_rs47815621
XRCC3_rs8615392
P2RX7_rs37511431
XRCC3_rs8615391
BMP2_rs2357682
IL10RB_rs28341672
IL18R1_rs114656601
RAC1_rs93741
0.0
0.1
0.2
0.3
0.4
relative importance
Boosted Trees
marker
IL2RA_rs112564972
IL18R1_rs114656601
miR.34b.c.promoter_r
ATM_rs1890372
IL10RB_rs28341672
RAC1_rs93741
0.0
0.2
0.4
0.6
relative importance
Random Forests
marker
IL6_rs1800795
IL1A_rs1800587
IL2RA_rs11256497
IL8_rs4073
ERCC1_rs3212948
IL10RB_rs2834167
SMAD1_rs11724777
BMP2_rs235768
miR.99a.promoter
RAC1_rs9374
0.00
0.05
0.10
0.15
0.20
relative importance
